# Praying correlates with higher quality of life: results from a survey on complementary/alternative medicine use among a group of Brazilian cancer patients

**DOI:** 10.1590/S1516-31802004000200005

**Published:** 2004-03-01

**Authors:** Eliana Sueco Tibana Samano, Patricia Taschner Goldenstein, Lia de Melo Ribeiro, Fabio Lewin, Edgar Santiago Valesin, Heloisa Prado Soares, Auro del Giglio

**Keywords:** Alternative medicine, Cancer, Quality of life, Medicina alternativa, Câncer, Espiritualidade, Oncologia, Qualidade de vida

## Abstract

**CONTEXT:**

The use of complementary/alternative medicine has been little studied in Brazil.

**OBJECTIVE:**

The purpose of this study was to determine the prevalence of complementary/alternative medicine use among a group of Brazilian cancer patients and correlate these findings with the patients' quality of life.

**TYPE OF STUDY:**

Descriptive.

**SETTING:**

Oncology Institute of the Faculdade de Medicina do ABC, Santo André, São Paulo, Brazil.

**PARTICIPANTS:**

100 cancer patients.

**PROCEDURES:**

The EORTC QLQ C-30 quality of life questionnaire was applied together with another questionnaire on the use of complementary/alter- native medicine.

**MAIN MEASUREMENTS:**

Use of complementary/al- ternative medicine and quality of life.

**RESULTS:**

89% of the patients had already used com- plementary/alternative medicine, 63% were currently using it and most of them (77.7%) believed in the efficacy of complementary/alternative medicine for their treatment. The type most used was individual prayer (77.5%). We found a significant association between believing in the efficacy of complementary/alternative medicine and praying (individually or in groups), in comparison with better scores on the functional (p = 0.001) and overall health (p = 0.001) quality of life scales. Multivariate analysis confirmed these findings regarding praying and also showed that believing in complementary/alternative medicine correlated significantly with functional and symptom quality of life scores.

**CONCLUSION:**

The prevalence of complementary/alternative medicine use in this group of cancer patients was high. Praying and belief in the efficacy of complementary/alternative medicine correlated significantly with an overall better quality of life, and therefore these practices should not be discouraged by physicians. New prospective studies should be conducted in order to better characterize the efficacy of such alternative therapeutic approaches.

## RESUMO

**CONTEXTO:**

O uso da medicina alternativa/complementar no Brasil é pouco estudado.

**OBJETIVO:**

Verificar a prevalência do uso de medicina alternativa/complementar por pacientes oncológicos e correlacionar os achados com a qualidade de vida.

**TIPO DE ESTUDO:**

Descritivo.

**LOCAL:**

Instituto de Oncologia da Faculdade de Medicina da Fundação do ABC.

**PARTICIPANTES:**

100 pacientes com câncer.

**PROCEDIMENTOS:**

Aplicamos questionário de qualidade de vida da EORTC QLQ C-30 *quality of life* e um questionário sobre o uso de medicina alternativa/complementar.

**VARIÁVEIS ESTUDADAS:**

Uso de medicina alternativa/complementar e qualidade de vida.

**RESULTADOS:**

89% dos pacientes já usaram medicina alternativa/complementar alguma vez, 63% estavam usando no momento do estudo e a maioria dos pacientes (77,7%) acredita na eficácia da medicina alternativa/complementar para o seu tratamento. O tipo de medicina alternativa/complementar mais utilizado foi a oração individual (77,5%). Encontramos associação significativa entre acreditar em medicina alternativa/complementar e a prática de orações (individual ou em grupo) com uma melhor qualidade de vida em relação à escala funcional (p = 0,001) e saúde global (p = 0,001). A análise multivariada confirmou estes achados, mostrando que rezar e acreditar na medicina alternativa/com- plementar se correlacionam significativamente com uma melhor qualidade de vida nas escalas funcionais e de sintomas.

**CONCLUSÃO:**

A utilização de medicina alter- nativa/complementar em nosso meio é fre- qüente em pacientes com câncer e a crença na sua eficácia e a prática de orações se correlacionaram significativamente com uma melhor qualidade de vida, de forma que tais práticas não devem ser desestimuladas pelos profissionais da área médica. Novos trabalhos prospectivos devem ser conduzidos para melhor caracterizar a eficácia destas práticas terapêuticas alternativas.

## INTRODUCTION

Complementary/alternative medicine can be defined as "drugs or therapeutic methods that have not been proven scientifically" and that therefore are not included within "the accepted orthodox biomedical framework of care for cancer patients".^1^ Despite concerns voiced by conventional practitioners in relation to its lack of scientific validity, the utilization of complementary/alternative medicine by cancer patients in different series has ranged from 7 to 64%.^2^ The reasons for the increasing use of complementary/alternative medicine include the limitations of conventional treatment, popularity of complementary/al-ternative medicine and the higher interest in natural treatments among cancer patients.^3^ In order to better evaluate the subjective effects of complementary/alternative medicine use in our patients, we prospectively surveyed 100 cancer patients treated at our outpatient facilities, regarding their pattern of use of com-plementary/alternative medicine, together with a quality of life evaluation.

## MATERIALS AND METHODS

For the purposes of this study, we defined complementary/alternative medicine as any form of therapy that is not included within the conventional treatment approach for cancer patients, including diets, psychotherapy, physical and movement-based therapies, mind/body treatments, spiritual practices, vitamins and herbs etc.^1^

This was an observational descriptive study. We included 100 non-consecutive patients who were being followed up at our outpatient clinic in the Oncology Institute of the Faculdade de Medicina do ABC. All patients were aged over 18 years and their cancer had been diagnosed at least two months before the survey. The patients gave informed consent for their inclusion. This study was approved by our Institutional Ethics Committee.

We employed the following questionnaires:

- General questionnaire: containing general medical information
- Complementary/alternative medicine questionnaire: we adapted the questionnaire developed by Richardson et al.^1^
- Quality of life questionnaire: we employed the European Organization for Research and Treatment of Cancer (EORTC) QLQ-C30 questionnaire, which had been already validated to be used in Portuguese.^4^

The statistical analysis was conducted through the NCSS 2000 software (Utah, United States).

## RESULTS

Our patient population consisted of 54 women and 46 men. The mean age was 59.2 years, ranging from 18 to 77 years, and most patients (90%) had not completed high school (Tables 1 and 2).

**Table 1:** Characteristics of the patient population studied

Characteristics	n
**Mean age**	59.2 years
**Gender**	
Female	54 (54%)
Male	46 (46%)
**Race**	
White	79 (79%)
Non-white	20 (20%)
**Educational level**	
Less than elementary school	68 (68%)
High school or higher	32 (32%)
**Religion**	
Catholic	71 (71%)
Other	29 (29%)
**Marital status**	
Married	61 (61%)
Non-married	39 (39%)
**Number of children**	
None	10 (10%)
One	5 (5%)
Two or more	85 (85%)
**Working status**	
Currently working	54 (54%)
Non-working	46 (46%)
**Income**	
< 2 times the minimum wage	53 (53%)
*> 2 times the minimum wage	40 (40%)
*Not disclosed	7 (7%)
**Way of traveling to the cancer center**	
Car	37 (37%)
Other ways	62 (62%)
**Performance status**	
100%	34 (34%)
70-90%	53 (53%)
< 60%	13 (13%)

*n = number;*

* *minimum monthly wage in Brazil is U$ 66.00 (at the time of writing).*

**Table 2:** Types of tumor and treatment received by the patients studied

Patient characteristics	n (%)
**Mean length of time since disease diagnosis**	27.43 months
**Type of tumor**	
Solid tumors	80 (80%)
Breast	26%
Prostate	26%
Colorectal	10%
Others	18%
Hematological tumors	20 (20%)
Present tumor status*	
Metastatic	34 (56.7%)
Non-metastatic	26 (43.3%)
**Previous surgery**	
Yes	63 (63%)
No	37 (37%)
**Previous radiation therapy**	
Yes	40 (40%)
No	59 (59%)
**Previous chemotherapy**	
Yes	59 (59%)
No	41 (41%)
**Presently on chemotherapy**	
Yes	59 (59%)
No	41 (41%)

*n = number of patients*

* *only the patients for whom we had accurate staging information at the time of questionnaire application are represented here.*

Most of the patients had used comple- mentary/alternative medicine previously (89%), believed in its efficacy for their treatment (77.7%) and were using complemen- tary/alternative medicine at the time of this study (63%). Patients who believed in the efficacy of complementary/alternative medicine reported that its use could improve their quality of life (90.4%), help in their treatment (77.7%) and cure their cancer (38%). Praying individually (96%) or in groups (94%) and spiritual surgery (93%) were the most frequently known types of complementary/alter-native medicine (Table 3). The types of com-plementary/alternative medicine that were most used among patients who had previously utilized complementary/alternative medicine (total of 89 patients) were: individual prayers (77.5%), group prayers (24.7%) and herbal therapies (23.6%).

**Table 3:** Patients' attitudes towards and use of complementary/alternative medicine (CAM)

	n (%)
**Belief in CAM**	
Yes	79 (79%)
No	16 (16%)
Don't know	5 (5%)
**Knowledge of someone who uses CAM**	
Yes	53 (53%)
No	47 (47%)
**Previous use of CAM**	
Yes	89 (89%)
No	11 (11%)
**Present use of CAM**	
Yes	63 (63%)
No	33 (35%)
No answer	4 (4%)

*n = number.*

We found that 83.7% of the patients who used complementary/alternative medicine did not inform their physicians about it. The most frequently reported reasons for this behavior were: 1) their physicians never asked (46.2%), and 2) they never asked and patients felt it was unimportant to tell their physicians about their use of complementary/alternative medicine (37.5%).

Believing in the efficacy of complemen-tary/alternative medicine correlated significantly with the overall health scale (p = 0.024), functional scale (p = 0.005) and with a better (i.e. lower) score on the symptom scale (p = 0.001). However, we could not find any correlation between current use of complemen- tary/alternative medicine and quality of life. Interestingly, the belief that complementary/ alternative medicine could cure cancer correlated significantly with the score on the overall health scale (p = 0.01). When we looked at the two most frequently used types of com- plementary/alternative medicine, we found that praying, either individually or in groups, correlated significantly with the scores on the overall health scale (p = 0.001) and functional scale (p = 0.001).

Multivariate analysis for each of the quality of life scales as dependent variables confirmed that praying was an independent statistically significant variable for the overall health and functional scales, whereas belief in complementary/alternative medicine and performance status were significant independent variables for the symptom and functional scales (Table 4).

**Table 4:** Multivariate analysis of scores on the different quality of life scales as dependent variables and several independent variables

	Symptom Scale		Functional Scale		Overall Health Scale	
	Regression coefficient	^p^	Regression coefficient	^p^	Regression coefficient	^p^
**Educational level**	0.2887799	0.728273	0.3409276	0.712276	0.4248409	0.682442
**Race**	17.80499	0.00576	-6.154978	0.379412	-1.787753	0.819744
**Income**	-1.692817	0.270773	1.501547	0.37901	2.554491	0.184195
**PS**	8.226206	0.024689	-13.34552	0.001298	-8.048486	0.076529
**Belief inCAM**	16.92553	0.007465	-17.58814	0.012159	-12.83455	0.099051
**Praying**	5.321531	0.38692	-14.26396	0.039371	-21.55294	0.006138

*CAM: complementary/alternative medicine, PS: performance status.*

## DISCUSSION

The use of complementary/alternative medicine is frequent among different cancer patient populations throughout the world, ranging in different series from 7 to 64%.^2^ Patients may use complementary/alternative medicine not only because of lack of hope in their conventional cancer treatment but also because they expect it may improve the success of the treatment.^3^ In fact, the expenditure on complementary/alternative treatment is increasing significantly and has reached U$ 34.4 billion per annum in the United States.^3^

The rate of complementary/alternative medicine use among our patients was high (63%) and near the upper limit of what has been reported in the literature.^2^ It was higher than the 43% rate that had been previously reported by Younes et al. in another Brazilian study.^5^ Unfortunately, however, that study was reported only in abstract form and there is a lack of detailed information regarding the complementary/alternative medicine types that were surveyed by the latter authors.

Attention is drawn to the fact that a large majority of our patients (83.7%) did not report their use of complementary/alternative medicine to their attending physicians. This was most probably due to the lack of interest among their physicians in finding out about it, which was manifested by the physicians' lack of questioning regarding the use of such practices among their patients.

Interestingly, whereas belief in comple- mentary/alternative medicine correlated significantly with the overall health scale of the EORTC QLQ-C30 quality of life instrument, the use of complementary/alternative medicine did not correlate significantly with any other of the quality of life scales of this questionnaire. Praying, however, either individually or in groups, correlated significantly with the overall health and functional scales of the EORTC QLQ C-30 quality of life questionnaire. Furthermore, most of these correlations persisted after adjustment for other important variables, as shown in Table 4.

## CONCLUSION

In conclusion, the use of complemen-tary/alternative medicine was frequent among our cancer population. The rate of use was probably underestimated by physicians, who may have had low interest in finding out about such practices among their patients. The use of complementary/ alternative medicine, probably through the patient's faith in its efficacy or enhanced spirituality while praying, correlates with higher quality of life. Therefore, physicians should not, in our view, discourage its use unless it can clearly be shown to be detrimental to their patients' health.
